# Outpatient Surgery for Rib Fracture Fixation: A Report of Three Cases

**DOI:** 10.7759/cureus.31890

**Published:** 2022-11-25

**Authors:** Daniel T DeGenova, Nolan P Schmitz, Jia Bao Lin, Travis J Jones, Benjamin C Taylor

**Affiliations:** 1 Orthopedic Surgery, OhioHealth Grant Medical Center, Columbus, USA; 2 Orthopedic Trauma, OhioHealth Grant Medical Center, Columbus, USA

**Keywords:** rib surgical fixation, rib plating, rib open reduction and internal fixation, outpatient rib fracture, same day surgery

## Abstract

Rib fractures are common injuries observed in trauma patients that will often heal without operative intervention. However, patients can infrequently have continued pain. Operative fixation of these symptomatic rib fractures has traditionally led to the patient requiring hospital admission for observation and pain control. The purpose of this study was to review three cases of outpatient rib fracture, open reduction and internal fixation (ORIF) surgery. Three patients with symptomatic rib fractures treated with ORIF at a single urban level one trauma center underwent outpatient same-day surgery. Pertinent demographic, clinical, radiographic, and surgical data were collected. All patients had decreased preoperative pain and no complications. This case series demonstrates that outpatient surgery for rib fracture ORIF can be performed safely in a select patient population. Additionally, it has similar efficacy as inpatient operative fixation with the main added benefit being decreased costs to both the patient and the healthcare system. We suggest that outpatient operative fixation of rib fractures should be considered for select patients.

## Introduction

Rib fractures are common injuries that account for 10% of blunt trauma hospital admissions [1]. Most rib fractures are treated nonoperatively, with nearly 80% of patients with multiple rib fractures showing evidence of healing based on computed tomography scans at three months post-injury [2,3]. Infrequently, non-operative management fails and leads to continued intractable pain [4].

There is increasing evidence about surgical treatment of rib fractures, particularly for flail chest injuries and for continued pain after failing nonoperative treatment [5,6]. Fowler et al. recommended surgical intervention for symptomatic rib fractures and those suffering from intractable pain [5]. A recent systemic review demonstrated operative fixation of rib fractures led to a signiﬁcant decrease in pneumonia, a decrease in mortality, and a decrease in hospital length of stay [6]. Surgical intervention for rib fractures has historically been associated with multiple days of hospital admission for observation after surgery [4,7].

There has been a recent surge of analyses regarding outpatient surgery for total joint arthroplasty [8,9], and more recently orthopedic spine surgery [10]. Meneghini et al. reported on the importance of optimizing preoperative, intraoperative, and postoperative protocols to ensure safe and successful outpatient surgery in total hip arthroplasty (THA) and total knee arthroplasty (TKA) [11]. Boddapati et al. mention patient selection as the most important factor when determining whether outpatient surgery is to be offered for three- and four-level anterior cervical discectomies and fusion (ACDF) procedures [12]. A major drive for outpatient surgery is the decreased total cost compared to inpatient surgery [13,14].

Although there appears to be a shift towards outpatient surgery for many other procedures, the authors are unaware of any reports of outpatient same-day surgery for open reduction and internal fixation (ORIF) of rib fractures. We describe the first three reported cases of ORIF of rib fractures performed as ambulatory same-day surgery.

## Case presentation

Case one

**Figure 1:**
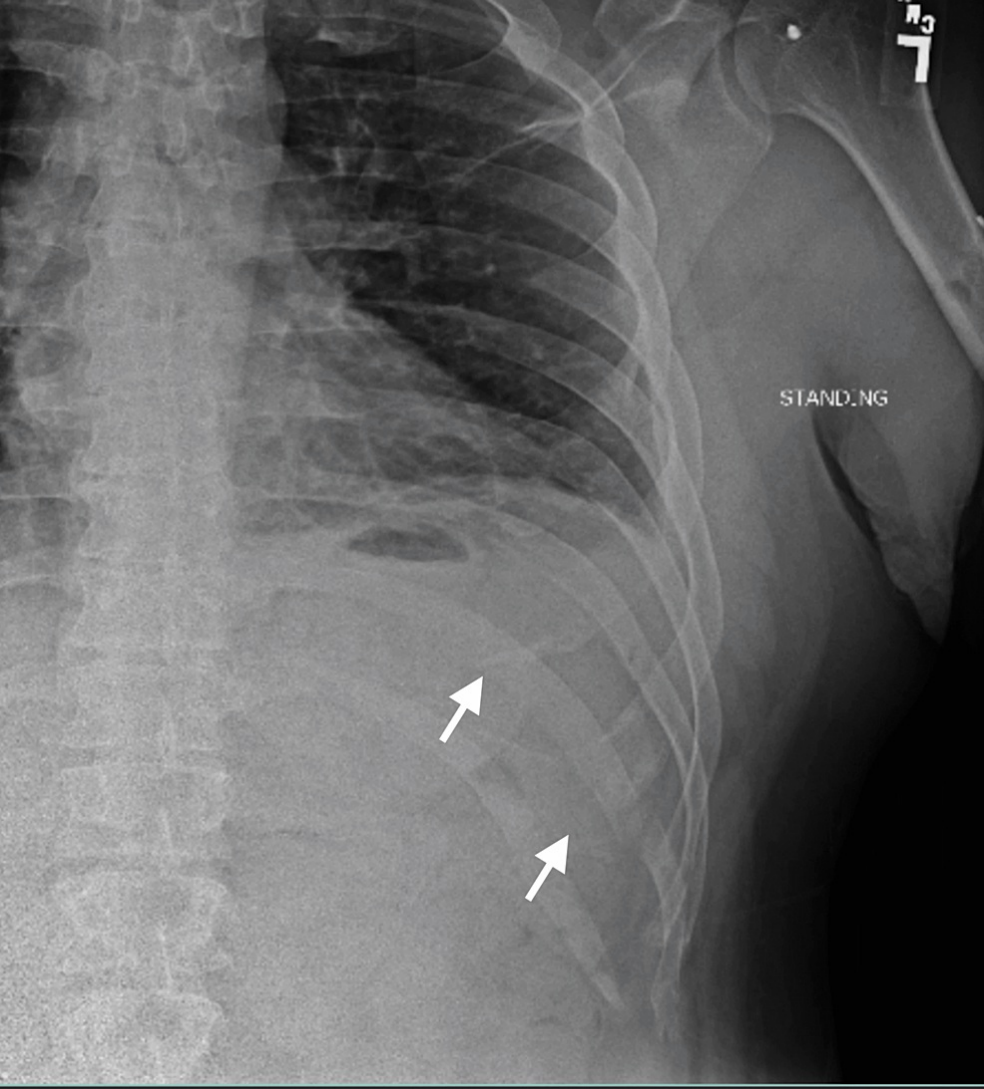
Anteroposterior preoperative chest radiograph The clinic-administered anteroposterior chest radiograph demonstrates posterolateral left-sided 10th and 11th rib fractures.

A 39-year-old male with no significant past medical history presented to the senior author’s clinic with continued left lower rib pain after multiple episodes of aggressive coughing 14 months earlier. Physical exam demonstrated full chest wall motion with tenderness over the posterolateral rib cage. Radiographs demonstrated posterolateral fractures of the left 10th and 11th ribs (Figure 1).

A trial of nonoperative treatment failed due to continued pain and hindrance to activities of daily living, and the patient elected to undergo surgical intervention. 

Surgical Procedure

The patient was brought to the operating room and positioned in the lateral decubitus position. A posterolateral muscle-sparing approach was used as previously described [15]. Rib 10 was freely mobile with abundant callus, whereas rib 11 was found to have a partial union of the external surface. The fracture sites were cleared of fibrosis tissue and callus formation. The medullary canals of the ribs were cleared of debris and fibrous tissue to allow for increased vascularization to the fracture site. Rib plating consisted of the RibFix Blu system (Zimmer Biomet, Warsaw, IN, USA), and a pure extrathoracic reduction and instrumentation were performed. No autograft or allograft was used. An indwelling pain catheter was placed to assist with postoperative pain control and a chest tube was not inserted. There were no perioperative complications. 

Postoperative Course

**Figure 2:**
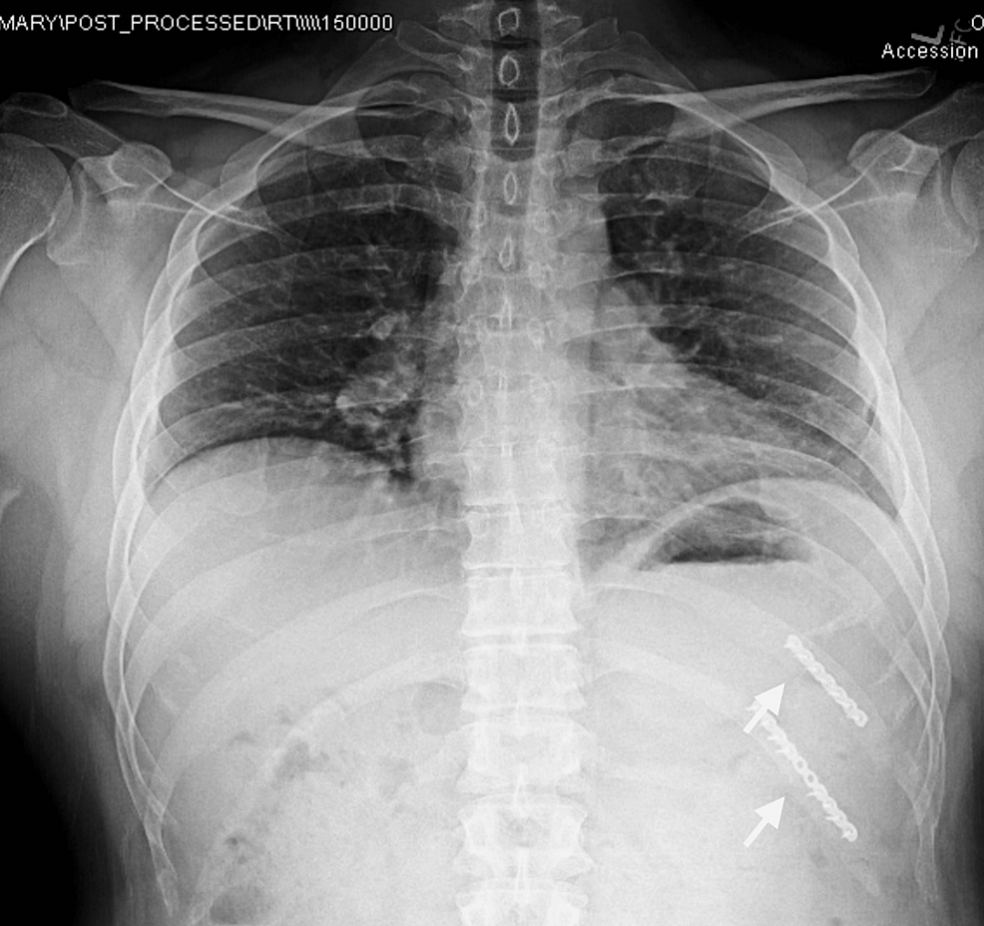
Immediate postoperative anteroposterior chest radiograph Immediate postoperative anteroposterior chest radiograph demonstrating operative fixation of the posterolateral 10th and 11th rib fractures without any signs of complications or pneumothorax.

Postoperative chest radiographs were obtained to assess the surgical results and to rule out any acute postoperative complications; none were found (Figure 2).

The patient did not require any supplemental oxygen. The patient was subsequently discharged to home on the same day as surgery after clearing post-anesthesia care unit (PACU) protocols with no restrictions and was able to perform daily activities as tolerated. The patient was instructed on how to remove their indwelling pain catheter on post-operative day three. 

The patient followed up at two weeks postoperatively with a well-healing incision and no complications. The patient was not taking any medications and did not fill his postoperative narcotic prescription. Physical examination showed full chest wall motion without restriction and ipsilateral shoulder and periscapular strength equal to the contralateral side. 

At 14 months postoperatively, the patient had no complications with full strength and range of motion but did have slight tenderness to palpation of the ribs anteriorly. The patient had returned to his previous level of employment prior to surgery. Radiographs at this final visit demonstrated plate fixation with appropriate positioning and no signs of displacement or loosening with evidence of bony union. 

Case two

**Figure 3:**
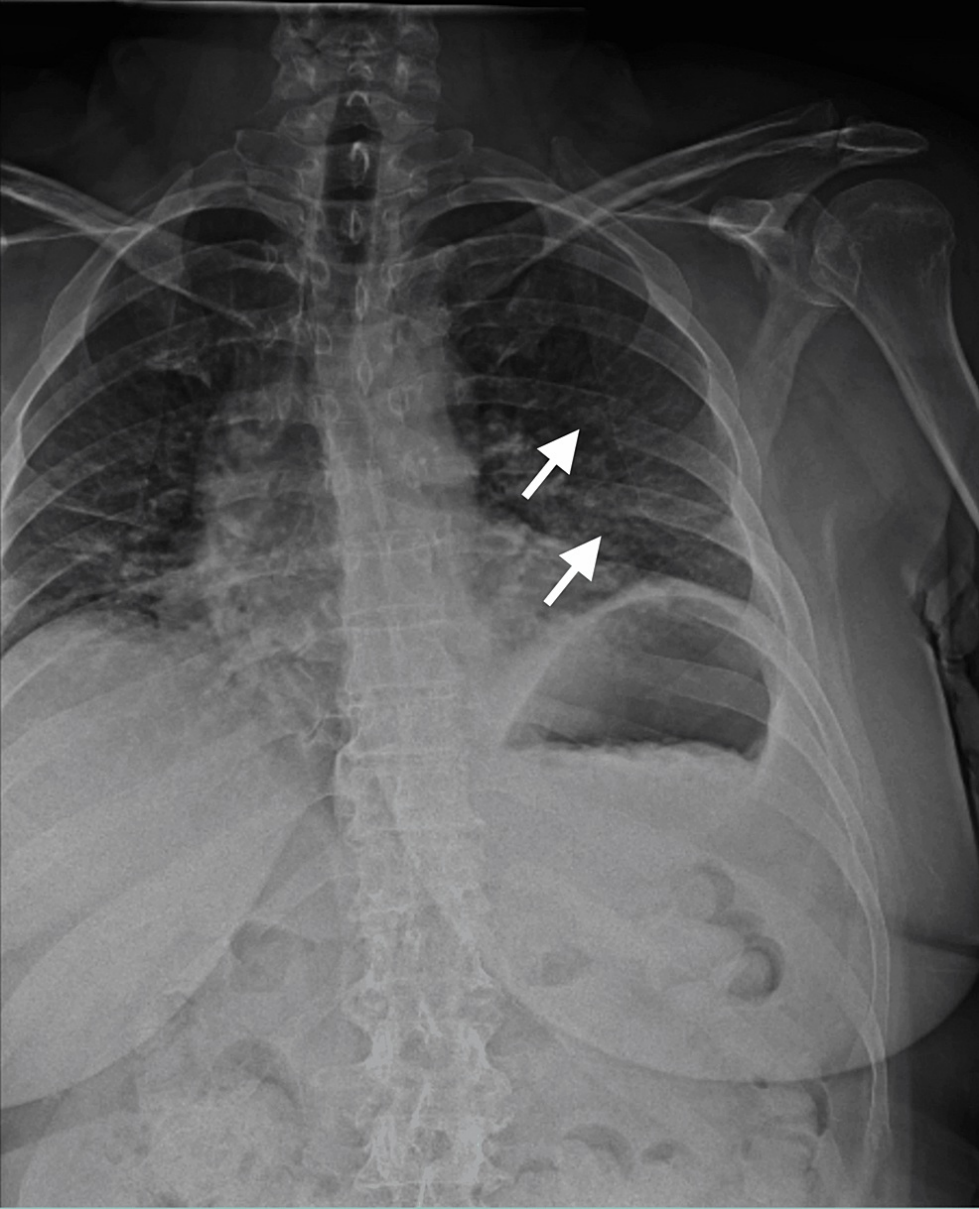
Clinic-administered chest radiograph The clinic-administered chest radiograph demonstrates a nondisplaced left fifth rib fracture and a displaced left sixth rib fracture.

A 61-year-old female presented to the outpatient clinic with left posterior rib pain after being forcefully shoved into a metal rack 10 months prior. There were no other associated injuries. The past medical history was positive for diabetes mellitus, hyperlipidemia, acute ischemic stroke, and hypothyroidism. A clinical exam demonstrated left posterior rib pain with limited ipsilateral shoulder range of motion and 4/5 ipsilateral shoulder strength. Radiographs demonstrated a nondisplaced fifth rib fracture and a displaced sixth rib fracture of the posterior rib cage (Figure 3). A CT scan confirmed these findings. The patient elected to undergo surgical intervention. 

Surgical Procedure

The patient was brought to the operating room and positioned prone. An incision was made over the center of the right fifth and sixth ribs using a posterolateral thoracotomy muscle-sparing approach. The fifth and sixth ribs were found to have minimal callus formation. The fracture sites and medullary canals were cleared of debris. A pure extrathoracic reduction and instrumentation were performed. No autograft or allograft was used. An indwelling pain catheter was placed; there were no intraoperative complications.

Postoperative Course

No postoperative complications were found on chest radiographs, and the patient did not require any supplemental oxygen immediately following surgery. The patient was subsequently discharged home on the same day as surgery after clearing PACU protocols with no restrictions and was able to perform daily activities as tolerated. The patient was instructed on how to remove the indwelling pain catheter on postoperative day three. 

**Figure 4:**
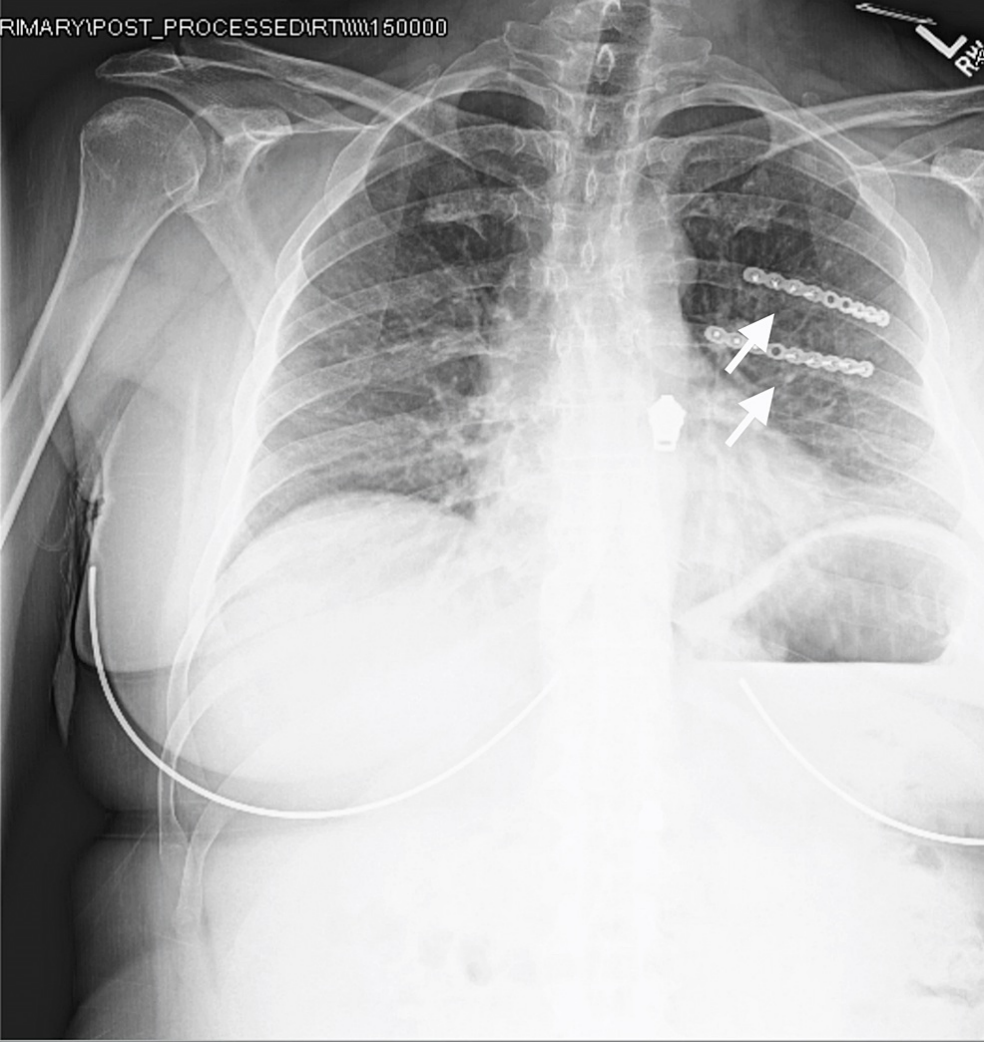
Anteroposterior chest radiograph Radiograph at final follow-up demonstrating intact hardware and union of the rib fractures.

At the three-week postoperative follow-up, the patient endorsed minimal pain that was decreased compared to pre-operative levels. At the follow-up visit four months postoperatively, the patient endorsed no pain, tightness, intercostal neuralgia, or seroma formation. Shoulder range of motion and strength improved to equal the contralateral side. At 30 months postoperatively, the patient was doing well and repeat radiographs demonstrated plate fixation with bony union (Figure 4). The patient was able to return to their previous level of employment prior to surgery.

Case three

**Figure 5:**
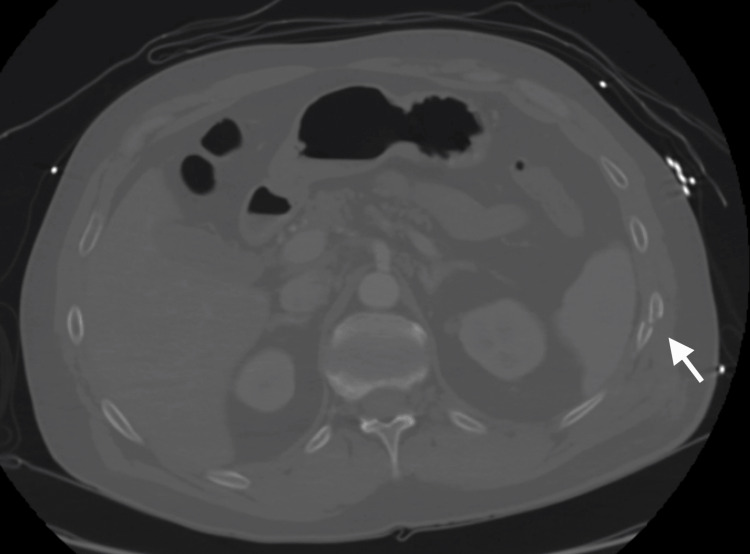
Axial computerized tomography scan Axial computerized tomography scan demonstrates a displaced left-sided posterolateral 10th rib fracture.

A 60-year-old male presented to the outpatient clinic with ongoing and significant left-sided chest pain after he fell 15 feet onto his back four months prior. Past medical history was significant for hypertension, controlled chronic obstructive pulmonary disease (COPD), and chronic kidney disease. A clinical exam demonstrated left posterolateral rib pain with a full ipsilateral shoulder range of motion and 5/5 ipsilateral shoulder strength. Radiographs and computerized tomography scans demonstrated a displaced 10th and 11th rib fracture (Figure 5). The patient elected to undergo surgical intervention after a trial of nonoperative treatment failed due to continued pain and hindrance to activities of daily living.

Surgical Procedure

The patient was positioned in the lateral decubitus position and a posterolateral muscle-sparing thoracotomy approach was used. Minimal callus formation was encountered. The fracture sites and medullary canals were cleared and an extrathoracic reduction was performed. The RibFix Blu system was used for implants and an indwelling pain catheter was placed. There were no intraoperative complications.

Postoperative Course

Postoperative chest radiographs did not demonstrate any postoperative complications, and the patient did not require any supplemental oxygen. The patient was subsequently discharged home on postoperative day zero with no restrictions and was able to perform daily activities as tolerated. The patient was prescribed a short course of narcotic pain medication to help decrease post-surgical pain, and the pain catheter was removed on postoperative day three.

**Figure 6:**
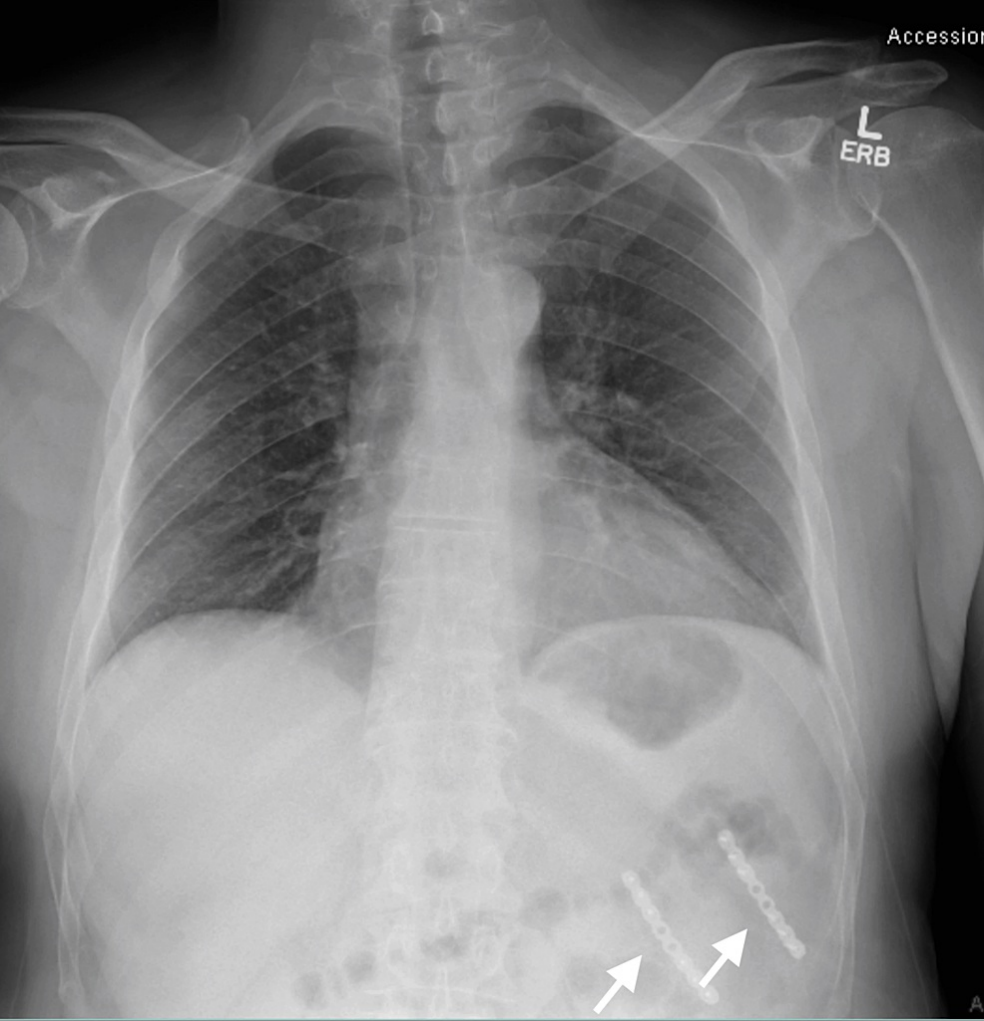
Anteroposterior chest radiograph The clinic-administered anteroposterior chest radiograph demonstrates operative fixation of the posterolateral 10th and 11th rib fractures at the most recent follow-up.

At the six-week follow-up, the patient endorsed minimal pain that was much improved from preoperatively. The pain was exacerbated only with movement and 4/5 ipsilateral shoulder strength was present. Six months postoperatively, the patient endorsed minimal pain that was much improved from preoperatively (Figure 6). 

## Discussion

Our retrospective case series successfully demonstrates that ORIF for rib fractures can be performed as an outpatient surgery. This study describes outpatient rib fracture fixation as an effective and safe treatment with the main added benefit being decreased costs to both the patient and the healthcare system. We advocate for careful and cautious patient selection. Additionally, our series demonstrated that ORIF for rib fracture fixation as outpatient surgery results in similar efficacy as inpatient operative fixation without increased incidence of intraoperative and postoperative complications, as well as reliable improvement in preoperative pain.

The majority of rib fractures are treated conservatively with a combination of multimodal pain control, pulmonary physical therapy, and bronchodilator inhalers with most healing without significant morbidity [16]; however, it is estimated that 5% to 10% of these patients will have continued pain that necessitates operative fixation [17]. There is increasing literature on the acute fixation of rib fractures, especially in the setting of flail chest injuries; however, there is not a universal or standardized postoperative protocol for patients undergoing rib fracture fixation [2,7]. Taylor et al. reported on 88 patients who underwent ORIF of acute flail chest injuries; the average hospital stay was 17 days and 55% of patients required ventilator treatment [18]. Numerous studies have shown the benefit of ORIF for flail chest injuries leading to decreased ventilator time, ICU, and hospital stay [5]. 

Several other studies have discussed rib fracture nonunion fixation or delayed fixation. This population may relate closely to our series as these patients are less acute and can be optimized for surgery. Traditionally, patients who undergo rib ORIF for symptomatic nonunion have spent at least one night in the hospital for observation [4,19]. DeGenova et al. recently published a series of 18 patients who underwent ORIF for rib fracture nonunion and the average hospital length of stay was three days [4]. Another series reported on 10 patients undergoing ORIF for rib fracture nonunion, where 40% of patients required chest tube insertion for lung parenchymal injury [19]. While it appears that the standard is observation overnight after rib fracture ORIF, the current study demonstrates evidence that in certain populations, rib fracture ORIF can be performed on an outpatient basis.

There is an abundance of literature discussing the value of careful patient selection in determining the ability to perform successful same-day surgery. The ideal patient for outpatient surgery is a relatively young and healthy individual with solid social support [8]. Courtney et al. retrospectively reviewed a cohort of patients who underwent TKA and THA and reported congestive heart failure, coronary artery disease, cirrhosis, and COPD were independent risk factors for a medical complication postoperatively [20]. The authors recommended against outpatient surgery for any patients with these risk factors [20]. 

We recommend careful patient selection for outpatient rib ORIF. In this series, each patient failed a trial of nonoperative treatment and did not have an operation in the acute setting. Open reduction and internal fixation (ORIF) in the acute setting is typically reserved for patients with flail chest or respiratory compromise [2]. All three patients in this series were able to be optimized in the outpatient setting before undergoing surgery. Additionally, the patients were non-smokers with minimal medical comorbidities.

One main benefit of outpatient surgery is the significant decrease in the cost to both the patient as well as the healthcare system. In a recent systematic review on outpatient total shoulder arthroplasty (TSA), it was demonstrated that inpatient TSA was 41.1% more costly than outpatient TSA even after removing inpatient-only charges [9]. Along the same lines, outpatient TKA has been shown to lead to savings of up to 33% compared to inpatient surgery [13]. Aynardi et al. found that there was a significant difference in cost between inpatient and outpatient THA with outpatient surgery saving nearly $7,000 [14]. Although the exact cost savings in our series is unknown, it can be extrapolated that it leads to a large reduction in healthcare costs to both the patient and the healthcare system. Due to this, we recommend outpatient surgery in the carefully selected patient to help reduce the healthcare cost burden. 

Lastly, many studies have reported equal or improved outcomes and functional results for patients undergoing outpatient surgery versus inpatient surgery. Snowden et al. compared 29 patients undergoing outpatient L5-S1 anterior lumbar interbody fusion to an inpatient group and found no difference in outcome scores between the groups at any time [10]. Additionally, the group found that the outpatient group had a lower total complication rate of 10.3% versus 15.1% in the inpatient group [10]. In the current series, each patient underwent ORIF for multiple rib fractures as outpatient surgery. None of the patients had intraoperative, perioperative, or postoperative complications. Each patient sustained union of their fractures with good functional outcomes demonstrated by 4-5/5 ipsilateral arm and periscapular strength. Patients also reported a decrease in preoperative pain. All patients in this series had a favorable outcome at an average of 16.6 months after their outpatient surgery. 

There are several limitations to this study. This study represents three retrospective case studies without a comparative group. With the nature of this study being a case report on three patients, it is a small sample. Further larger studies are needed to increase the validity of these findings. With the sample size being this small, the conclusions that can be drawn are limited, but this study adds to the paucity of literature on the outpatient treatment of rib fractures with ORIF. 

## Conclusions

Most rib fractures can be successfully treated nonoperatively with few complications. Infrequently, patients have continued pain after these fractures. Traditionally, when patients undergo open reduction and internal fixation for rib fractures, they have been admitted to the hospital for pain control and observation. This case series demonstrates that outpatient surgery for rib fracture via open reduction and internal fixation can be safely performed. Additionally, it has similar efficacy as inpatient operative fixation with the main added benefit being decreased costs to both the patient and the healthcare system. We suggest that outpatient operative fixation of rib fractures should be considered for select patients.
